# Introducing youth to clinical research: Development and pilot implementation of a children’s activity book

**DOI:** 10.1017/cts.2024.678

**Published:** 2025-01-03

**Authors:** Renee Cadzow, Andy Strohmeier, Erin Carnes, Teresa Quattrin

**Affiliations:** 1 Department of Epidemiology and Environmental Health, School of Public Health and Health Professions, University at Buffalo, Buffalo, NY, USA; 2 Clinical and Translational Science Institute, Jacobs School of Medicine and Biomedical Sciences, University at Buffalo, Buffalo, NY, USA; 3 Department of Pediatrics, Jacobs School of Medicine and Biomedical Sciences, University at Buffalo, Buffalo, NY, USA

**Keywords:** Children, clinical research, clinical trials, diversity, education, special populations

## Abstract

**Introduction:**

Children continue to be an underrepresented population in research and clinical trials due to difficulties encountered in recruitment, assenting, and retention processes. “Sofia Learns About Research” is a children’s activity book that introduces youth to clinical research and basic elements of clinical trials.

**Methods:**

Development of the activity book began in 2016, with publication of the first paper version in 2017 and an online version adapted for computer and tablet users in 2019. In 2019, we developed internal review board-approved pre/post surveys with five statements (written at ≤ 3^rd^-grade level) reflecting key concepts covered in the book. Participants were asked to indicate whether they agreed, disagreed, or were not sure about each of the statements and if they would ever want to be part of a research study. Preliminary analyses included descriptive statistics and cross-tabulations with chi squares.

**Results:**

Despite delays in dissemination and outreach due to the COVID-19 pandemic, we obtained feedback from over 170 diverse persons across a spectrum of communities and community partners. After book exposure, more participants knew that both children and parents have to assent/consent and that participants can withdraw from a study at any time.

**Conclusions:**

The book is an important advocacy tool with a long-term aim of increasing children’s knowledge and awareness about clinical research, ultimately leading to enhanced participation in clinical research and trials.

## Introduction

Child participation in clinical research is challenging due to multiple complex barriers. Most commonly, a lack of awareness and understanding of research among youth and their parents/guardians precludes children from participating in clinical trials [1]. Also, many marginalized and/or minoritized communities have a general distrust of the healthcare sector; and clinical research is particularly difficult to trust, in part, due to historic atrocities. Building relationships and trust over time between researchers and their institutions and working closely with community advocates will support recruitment and retention [2]. Proper communication of the risks and benefits of research to children and their parents is not easy and can be met with hesitancy, often because the potential benefits are not clear, but also due to cultural and language barriers. Therefore, it is not surprising that pediatric trials are often small, lack diversity, and drug approval for pediatric indications is often the result of extensive testing only in the adult population [3].

In 2015, we conducted an informal search for informational materials tailored to youth on research participation and recruitment and found very few resources. Those available were often pharmaceutical industry-sponsored and related to drug trials for specific illnesses or conditions. Our review also revealed that most materials targeted parents and were therefore not written for a child audience. Studies suggest, though, that parents and children desire materials that support joint decision-making [4]. Further, Graves and Sheldon provided a comprehensive assessment of working specifically with African American children for research, but many of their lessons learned apply to other minoritized and/or historically marginalized populations [5]. They frame research as a process involving many systems and structures that intersect. Recommended strategies include meeting with and having open discussions with potential participants about research, affirming their existing knowledge and expertise, and working with trusted partners (community centers, schools, afterschool programs, churches) [5]. In a study of assets and challenges in recruiting children and families in obesity-related research, researchers identified challenges related to “comfort and trust with research” and “awareness and understanding of the study.” Among the recommendations listed were using multiple modes of media, ensuring materials are responsive to different levels of health literacy, and partnering with trusted local organizations [6]. Finally, a meta-analysis of studies using multiple types of media (animations, videos, etc.) compared to the standard printed participant information sheets found that multimedia approaches were more effective at recruiting children and youth to randomized clinical trials [7].

This project began in 2016 as part of the University at Buffalo Clinical and Translational Science Award. Among the objectives was to increase the participation of “special populations” in research. In the process of developing our strategy for outreach to pediatric populations, we sought to address the gap in materials that explained the research and clinical trial process to children and their families in a clear, fun, and interactive way [8–13]. A multigenerational team comprised of a pediatrician (CTSI Integrating Special Populations Core Lead), an anthropologist (CTSI Integrating Special Populations Task Leader), and a pre-medical student (research assistant) developed a book with the goal of being accessible to children and families of various cultures, learning, and literacy levels. We built on the experience of the lead authors in pediatrics and family-based obesity research, a review of the literature, and an exhaustive web search of child and family-friendly informational tools about research (conducted in 2016). Herein we describe the process of developing and illustrating “Sofia Learns About Research,” as well as our initial efforts toward quality improvement and ultimately attempting to measure the book’s impact on research knowledge and interest in participation.

## Materials and methods

The initial phases of book development and implementation, inclusive of the needs assessment, development, and quality improvement, were not conceptualized as research. We developed the book as an educational tool and then obtained feedback from various audiences in an effort to improve the book and inform dissemination and engagement strategies. In 2019 we established a research protocol that was approved by our internal review board (IRB), enabling us to collect and analyze the feedback provided in several settings.

### Needs assessment

Before drafting the story, the team explored community perceptions of research and involvement of children in research through community conversations with school nurses, community health workers, and a local child health-related coalition (Table 1). These constituencies were selected due to their close interface with children and their families around issues of health, well-being, and clinical research. Themes of discussions included a distrust of research, the perception that it always involves testing a drug, the worry of being treated like guinea pigs, and the feeling that it does not provide any benefit to the participant. These concerns, however, were balanced by some positive perceptions, including a general feeling that research can lead to better health and illness treatments. Of note, this feedback was elicited entirely from adults who work with children, not the children themselves. We included feedback from children after we completed the first draft of the story in late 2016, as described below.

**Table 1.** Needs assessment community conversations about research

Date	Population	Participants
August 9, 2016	Members of Healthy Baby Coalition at United Way (representatives from maternal/child health nonprofit, healthcare, and government agencies)	∼ 30
September 19, 2016	School-based Clinic Nurse Practitioners	9
January 24, 2017	Community Health Workers (at a monthly “Chew and Chat”)	∼ 30
March 7, 2017	School Nurses	∼ 40

The aforementioned existing literature and the themes that emerged in these conversations with adults informed the topics to be covered in the book. This included clear involvement of the whole family in decision-making, the consent and assent process, the various types of data that are collected (not just drug testing trials), the ability to leave the study at any time, and the relevance of the research to the participant and/or the wider community. Given the variety of research (behavioral, clinical, quantitative, and qualitative) associated with the common illness of asthma, we selected asthma as the example used in the book.

### Development and quality improvement

#### Development

With the identified themes and health conditions, our team constructed a story of a young girl going to the doctor and being informed about and invited to participate in a study about her asthma. We researched names to enhance the relatability and accessibility of the story for the characters and identified “Sofia” and “Michael” (Sofia’s little brother) as cross-culturally common names. In the story, Sofia has conversations that gradually build her knowledge and trust, leading her to understand what a clinical trial means and why she and her family may want to consider enrolling in it. Sofia and her family are introduced to the basic concepts of clinical research and clinical trials. The story is interspersed with activities like word searches, crack the code, mazes, and other “research” style games to appeal to different types of learners and different age groups. The story was completed and then revised after applying the Flesch-Kincaid Grade Level reading tool in Microsoft Word to ensure that it did not exceed a third-grade reading level.

In late 2016, the initial draft of the story, without illustrations, was shared with a group of children and parents from diverse backgrounds, some of whom had previously participated in clinical and/or behavioral research, and some of whom had a chronic condition. Children and their parents reviewed the book, tried the activities, and provided their feedback related to several of the elements of the story, including ideas for illustrations and additional activities. Among their suggestions were adding a glossary of terms, introducing both of Sofia’s parents (only the father was present in this draft version), and having Sofia’s younger brother Michael integrated more into the story. Children also proposed having Sofia and Michael transform into a more exciting duo, which led the team to the idea of Sofia and Michael becoming research superheroes called the “Research Rangers.”

The edits suggested by the children were incorporated into the final version of the book. A professional cartoonist with experience producing health education resources illustrated the book. This was an iterative process where we provided our ideas of how the concepts could be illustrated and she drafted several versions to review and revise. Illustrations show that the conversation about research is inclusive of the parent and the children [4]. The clinicians and researchers discuss the study with both Sofia and her father and address her brother Michael’s questions as well. In this way, they establish that it is not just the parents’ decision, but also Sofia’s. This is confirmed by Sofia’s dad, when he says, “It’s nice to meet you, too. *We* would like to hear more about it. Thank you.” A conversation about research follows and is a collaborative discussion between Dr Q, Sofia, her brother Michael, and her father. Additionally, the conversation around consent and assent to participate in a study highlights that it is a decision of both the parents and the children. This emphasizes that a parent cannot enroll a child in a study without the child’s assent to participate.

Also informed by the literature [5,6], the importance of building a relationship and trust is highlighted. Sofia makes a reference to trust in the book. She identifies characteristics of her interaction with the researcher that contribute to her trust (she answers her questions and is friendly), but she still has concerns related to the fact the study will take time away from her usual activities with friends and what she would tell her friends about it. Importantly, Sofia feels comfortable expressing her concerns, and Dr Q responds that they will do their best to be responsive to her schedule and her needs and reassures her that she is able to leave the study at any time. Building on this, the gameboard illustration at the end of the book shows how Sofia is able to continue with her usual activities (like going to the movies, out for pizza, and having sleepovers) while also going to the doctor for blood work, starting a new medicine, and taking surveys. She has an unanticipated benefit of learning more about research and applying it to her schoolwork as well.

We opted to intersperse the games throughout to allow the readers to apply the skills as they learn them (e.g. identifying new words in a word search and crossword puzzle and practicing observation skills in the “find the difference” activity). For children who do not yet understand the research process and are not developmentally capable of providing their assent, this also provides an activity to do while older children and parents discuss a study and possible participation.

**Figure 1.**
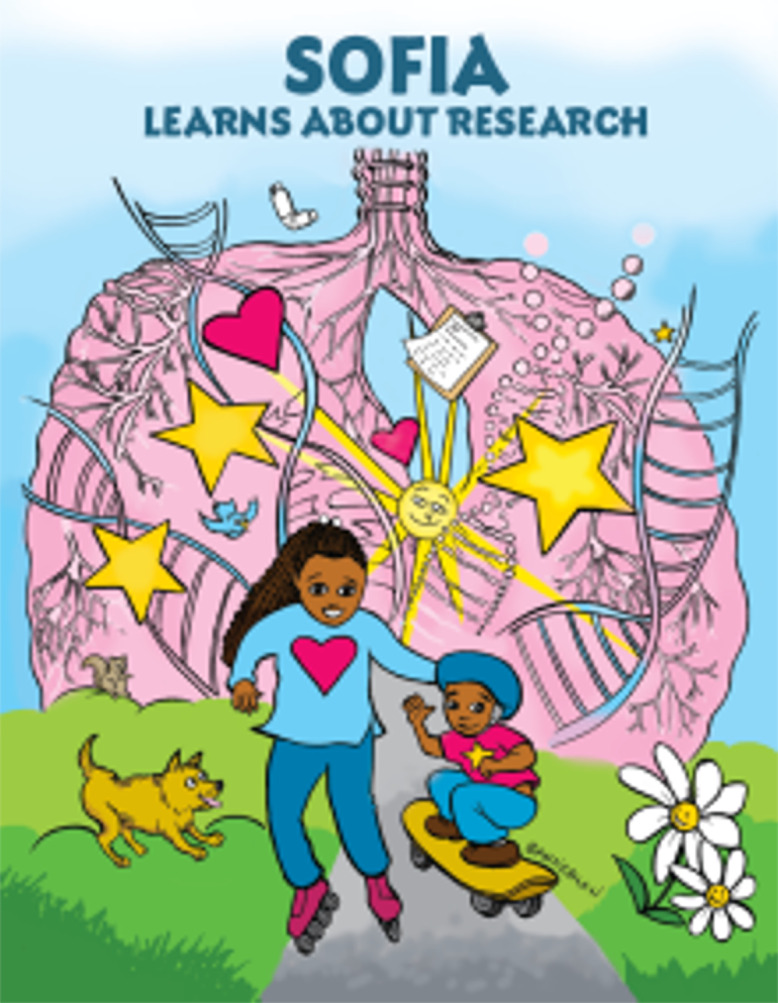
Cover of Sofia Learns About Research.

Finally, consistent with the book functioning as an activity book as well as a learning tool, we opted for the book cover in color and all illustrations in black and white to promote use as a coloring book. This also made it accessible to children of many backgrounds, as they could color the characters in ways that matched their preferences or identities (Fig. 1).

#### Quality improvement

The first version (PB-E: Paper Book English version) was completed in July 2017 (Table 2). We printed several hundred copies of the book and began sharing in afterschool settings, schools, and clubs (e.g. Girl Scout Troops) along with a group pre/post evaluation survey to be implemented by the onsite facilitator of the book reading (e.g. program staff). Questions asked before reading included: grade level of children in the group, “what do you think of when you hear the word “research?” and “How many of you think you would ever want to be part of a research study?” Questions asked after reading and completing book activities included “How many of you think you know a little more about research than you did before?” “How many of you think you would ever want to be part of a research study?” “What are your favorite parts of the book?” And “What do you think could be changed or added to make the book better?” Program staff provided the book authors with summaries of children’s responses, including that they learned about research and that their favorite parts of the book were the activities: maze, crack the code, and word search. A limitation of this approach was that the facilitators of the book were program staff, not the book authors. While guidance for the book readings was provided, we cannot confirm that the reading of the book and collection of the feedback was consistent across sites.

**Table 2.** Iterations of the Sofia Learns about Research book rationale

Sofia Learns about Research Version	Year	Rationale
PB-E: Paper Book in English with color cover and black and white pages; also downloadable as PDF on UB CTSI website	2017	Address the gap in child-friendly research informational tools Extend access to the informational tool to partners outside the region and to children and families more broadly
PB-S: Paper Book in Spanish and available as a downloadable PDF on our website	2018	Extend access to the informational tool to Spanish speaking families
PB-A: Paper Book in Arabic (with updated illustration of the focus group to include a young woman with a hijab) and available as a downloadable PDF on UB CTSI website	2018	Extend access to the informational tool to Arabic speaking families (3^rd^ most common language in the region)
DI-E: Digital, interactive English version (ability to color pictures and write on the pages online)	2019	Provide interactive online experience. This allowed for individuals to access and fully experience it without the need to print it.

In February 2019, the researchers conducted the first in-person book readings (PB-E) in two 5^th^-grade classes at a local charter elementary school, reaching approximately 50 children. We split each reading session into two days and facilitated active discussion with the children during each session. Further supporting quality improvement of book implementation, we used our notes from our reading sessions and youth’s comments in the letters that the children sent to us after our visit to understand the impact of the book reading on children’s knowledge. The children were familiar with research in general, though most examples related to doing internet or literature searches for information. The topic of asthma was familiar; almost all students knew of someone with the condition. Children expressed that they would like to conduct research on individual health issues like ADHD, autism, and craniofacial differences, as well as more global health issues like rainforest life and global warming. Teachers and children liked the activities in the book, citing that the variety made it fun for everyone. Below are examples of comments abstracted from thank you letters the children wrote:

*“Thank you for coming to our fifth-grade classroom and reading us the story about Sofia. My favorite part was when we did the cross-word puzzle. I learned that people are studying asthma to try and make better medicine and cure it.”*

*“I really liked the story about Sofia and I want to stop autism, eye loss and hearing loss so thank you for sharing the story about Sofia that really helped me understand and learn some new things and words.”*

### Evaluation research

Informed by this feedback, individual-level evaluation tools were subsequently developed to assess participants’ perception of research after reading the book (post evaluation only) as well as the participant’s change in knowledge and interest in research (pre/post evaluation). The post evaluation-only approach was determined to be exempt by our Institutional Review Board in March 2019. Book readings with pre/post evaluation were subsequently approved by the IRB with a waiver of parental permission and assent as it presented no greater than minimal risk and the information and activities presented in the book are concepts that youth would encounter during the curriculum. The questions in the pre/post surveys were similar to the types of questions that teachers would pose to their pupils in daily classroom instruction. The approved IRB protocol allowed for variation in the book reading implementation [PBE or Digital Interactive English (DI-E) version, led by classroom teachers, researchers/authors, or the participants themselves]. It also allowed flexibility for the length of the reading, number of breaks, and the nature of activities occurring in those breaks (discussion, application of concepts, games, etc.) in order to be responsive to student age, level of cognition, and attention spans.

The post- evaluation approach was piloted in June 2019 as a link to a survey printed on the back of the book (PB-E) along with a QR code. The survey queried whether the respondent was the child or the parent/caregiver of the child, the child’s age, gender, how much they knew about research before reading the book (a lot, a little bit, nothing at all), whether they think they know a little more about research now that they have read the book (yes/no/not sure), whether they would have ever thought about being in a research study before reading the book, whether they now think they would ever want to be part of a research study, what their favorite part was, what changes they suggest, and what other books about Sofia they would like to see. Over 100 copies of the book with the link to the survey were distributed but given minimal response, we discontinued this approach.

For the pre/post evaluation, we developed five statements (written at ≤ 3^rd^ grade level) reflecting key concepts covered in the book (Table 3). Participants were asked to indicate whether they agreed, disagreed, or were not sure about each of the statements and if they would ever want to be part of a research study. In the post survey, we also asked about their favorite part of the book, what changes they suggested, and their age.

**Table 3.** Pre/Post reading evaluation survey questions

Statement	Answer options
Only children who are sick can participate in research studies. (False)	Agree/Disagree/Not Sure
Research can help scientists and doctors make better medicine. (True)	Agree/Disagree/ Not Sure
If you are in a research study, you always have to give a little bit of your blood. (False)	Agree/Disagree/ Not Sure
To be in a research study, children and their parents or guardians both have to want to do it. (True)	Agree/Disagree/ Not Sure
Once children and their parents agree to be in a research study they have to stay in it until the end. (False)	Agree/Disagree/ Not Sure
(Pre) Do you think that you would ever want to be part of a research study? (Post) Now that you have read the book and done some of the games in it, do you think you would ever want to be part of a research study?	Yes/No/Not Sure
(Post) What was your favorite part of the book?	Open text
(Post) What do you think could be changed or added to make the book better?	Open text
(Post) How old are you?	Numerical
(Post) Are you a girl, boy or do you prefer not to answer?	Girl/boy/prefer not to answer

During our initial dissemination and evaluation of the book’s impact on research knowledge and interest in research participation, we identified the need to translate the book into other common languages and to create an online version of the book to access via the institution’s website and to share outside of our region. Table 2 outlines the various iterations of the book responsive to feedback from participants and community partners. The Spanish (PB-S) and Arabic (PB-A) version translations (the most common languages spoken in our region after English) were completed in October 2018 in partnership with the International Institute of Buffalo. These versions have not yet been included in the evaluation of the book; they have largely been made available as information-only. This paper presents implementation and evaluation of PB-E and the DI-E version.

### Outcome/impact evaluation

The global pandemic slowed our in-person dissemination and evaluation efforts substantially; however, we were able to share the DI-E in two ways during this time. Between May and June 2020, while schools were entirely virtual, our charter school partner incorporated the DI-E version in their science curriculum and included the pre/post IRB-approved survey to measure impact. The teacher provided an introduction, explained the purpose of the book, and provided opportunities for the students to ask questions.

Also, we shared the DI-E version and the pre/post survey with 550 members of the Buffalo Research Registry who had expressed interest in children’s related research. This method of implementation was entirely passive and involved no additional facilitation by teachers or researchers. We received feedback from children and/or their parents/guardians through this approach.

In summer of 2022, when many organizations were meeting in person again, we held book readings (PB-E) at the children’s museum science camp and at a local community center youth program. The readings at the science camp were structured similarly to our initial charter school sessions, where the researchers read and paused to ask questions and elicit feedback. The book reading was completed in two sessions to hold student attention and ensure time to complete activities. After considering these experiences, evaluation results, and discussions with teachers and program staff, the researchers adapted the book reading with some additional discussion prompts to maximize interaction and engagement with the concepts. With 6^th^–8^th^ grade youth at the community center, we discussed research, reviewed the book, and then prompted them to share some ideas about how they would recruit someone to participate in a research study. This, along with the evaluation survey, helped us observe the youths’ understanding and general attitudes about research.

## Preliminary results

Between 2020 and 2023, 170 youth and adults participated in either in-person or online exposures to the book from 25 Western New York zip codes, of which 64.6% belong to areas designated as underserved and/or high poverty. Out of 170 participants exposed to the book, 145 answered a pre survey and 95 took a post survey. Due to some technical issues, we were not able to match all pre-surveys with the same individuals’ post surveys. Eighty-nine (89) of the pre/post surveys could be reliably linked, had the respondent age, and were therefore used in these preliminary analyses. There were 31 children ages 5–9, 39 children ages 10–13, and 19 adults ages 30–68. The adults who completed the surveys were parents/guardians of children who accessed the book online after receiving it through the research registry.

**Figure 2.**
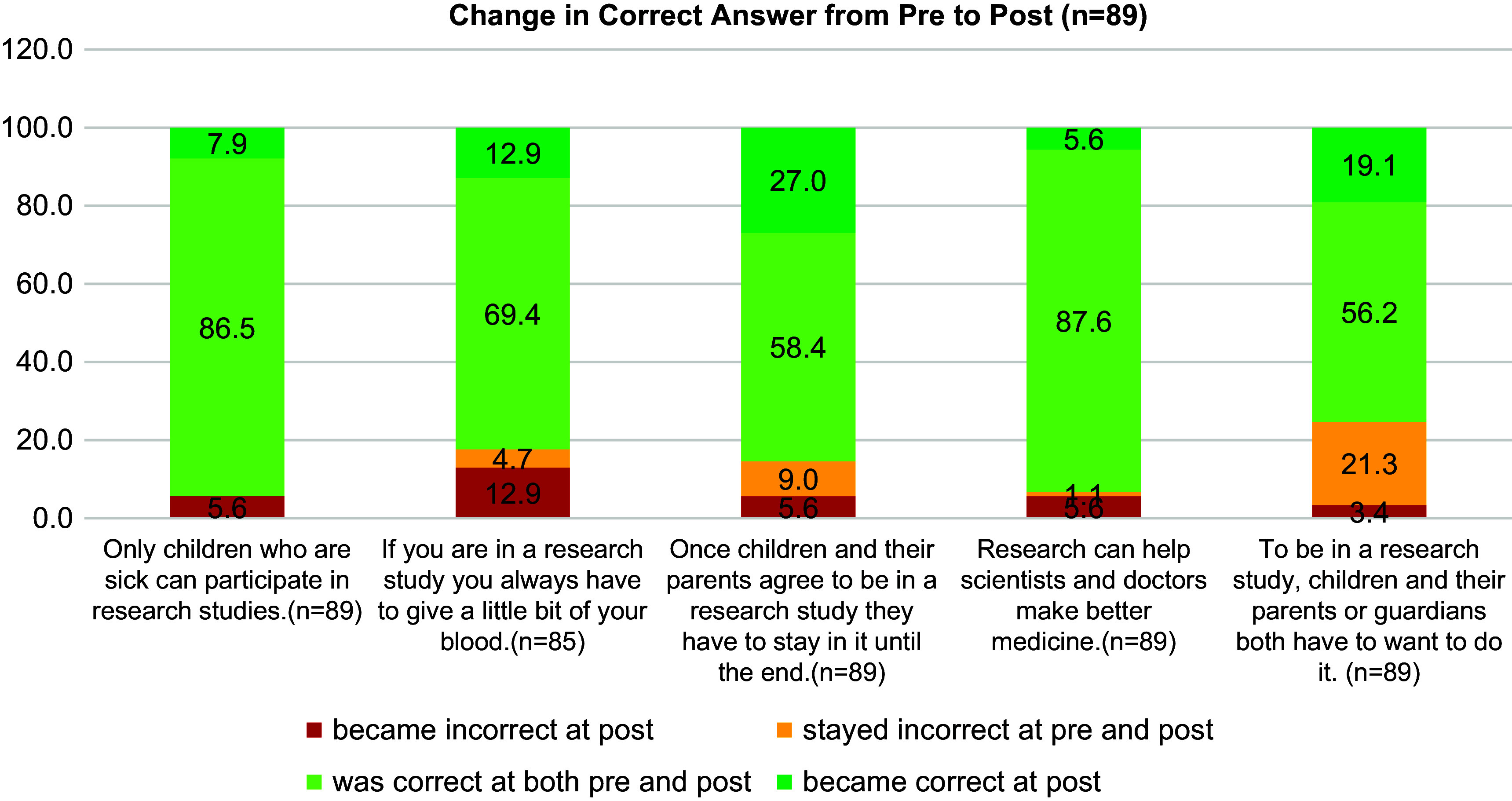
Change in correct answer from pre to post reading of Sofia book.

Analyses presented here include descriptive statistics and cross-tabulations with chi squares analyzing differences by age group and book modality (online vs. in-person) in change and knowledge and intention to participate in research from pre to post book exposure. Overall, most participants already knew that it was not true that “only people who are sick can participate in research studies” and that it was true that “research can help doctors make better medicine.” This is shown by 86.5% and 87.6% answering this statement correctly at both pre and post (Fig. 2). An improved understanding was shown post reading regarding the facts that both children and parents have to assent/consent and that participants can withdraw from a study at any time. This is shown by 27% and 19% more participants, respectively, answering these questions correctly after having first answered them incorrectly (Fig. 2).

Change in knowledge varied by age group. There were three statements for which the youngest respondents answered incorrectly at post-read after answering correctly at pre-read (statements 1, 3, and 5). Interestingly, all these statements are false. On the other hand, several children in the 5–9 and 10–13 age groups answered correctly at post-read after answering incorrectly to the statement “to be in a research study, children and their parents or guardians both have to want to do it.” This suggests that participants did learn that it was a joint decision. Similarly, the understanding that you could leave a study before completion improved from pre- to post-read among the children. The differences from pre to post by age group were statistically significant (*p* = 0.025 and *p* = 0.002, respectively) for statements 4 and 5 (Table 4).

**Table 4.** Change from pre to post by age category (*n* = 89)

		Became incorrect at post	Stayed incorrect at pre and post	Was correct at both pre and post	Became correct at post	p-value
Only children who are sick can participate in research studies.	age 5–9 (*n* = 31)	**4 (12.9%)**	0 (0.0%)	24 (77.4%)	3 (9.7%)	0.246
	age 10–13 (*n* = 39)	1 (2.6%)	0 (0.0%)	35 (89.7%)	3 (7.7%)	
	age 30 and older (*n* = 19)	0 (0.0%)	0 (0.0%)	18 (94.7%)	1 (5.3%)	
Research can help scientists and doctors make better medicine.	age 5–9 (*n* = 31)	3 (9.7%)	1 (3.2%)	24 (77.4%)	3 (9.7%)	0.362
	age 10–13 (*n* = 39)	2 (5.1%)	0 (0.0%)	35 (89.7%)	2 (5.1%)	
	age 30 and older (*n* = 19)	0 (0.0%)	0 (0.0%)	19 (100.0%)	0 (0.0%)	
If you are in a research study you always have to give a little bit of your blood.	age 5–9 (*n* = 29)	**7 (24.1%)**	2 (6.9%)	16 (55.2%)	4 (13.8%)	0.107
	age 10–13 (*n* = 39)	**4 (10.8%)**	2 (5.4%)	25 (67.6%)	**6 (16.2%)**	
	age 30 and older (*n* = 19)	0 (0.0%)	0 (0.0%)	18 (94.7%)	1 (5.3%)	
To be in a research study, children and their parents or guardians both have to want to do it.	age 5–9 (*n* = 31)	1 (3.2%)	11 (35.5%)	12 (38.7%)	**7 (22.6%)**	0.025
	age 10–13 (*n* = 39)	2 (5.1%)	8 (20.5%)	21 (53.8%)	**8 (20.5%)**	
	age 30 and older (*n* = 19)	0 (0.0%)	0 (0.0%)	17 (89.5%)	2 (10.5%)	
Once children and their parents agree to be in a research study they have to stay in it until the end.	age 5–9 (*n* = 31)	**4 (12.9%)**	3 (9.7%)	10 (32.3%)	**14 (45.2%)**	0.002
	age 10–13 (*n* = 39)	1 (2.6%)	5 (12.8%)	25 (64.1%)	**8 (20.5%)**	
	age 30 and older (*n* = 19)	0 (0.0%)	0.0%	17 (89.5%)	2 (10.5%)	

Eighty-one participants answered the question “Do you think that you would ever want to be part of a research study?” Of the 9 who answered “no” at pre-read, 5 still answered “no,” 1 answered “yes” and 3 answered “not sure” at post-read, indicating a shift in the desired direction. Of the 32 who answered, “not sure” at pre-read, 8 answered “yes,” 21 answered “not sure,” and only 3 answered “no” at post-read. Of the 29 who answered “yes” at pre-read, all but two answered “yes” at post-read; the other two answered “not sure.”

Change in intent to participate in research varied by modality (online vs. in-person). Among those who viewed online, all of those who answered “yes” to “Do you think that you would ever want to be part of a research study?” pre-reading still answered “yes” post reading. Also, 3 of those who were “not sure” and one of the 5 who answered “no” changed their answer to “yes” post reading. In the in-person group, the change in willingness to participate in research was slightly different: 57% of those who answered “no” moved to “not sure” and 28% of those who were “not sure” moved to “yes” at post-read. However, 22% of those who were “yes” at pre-read moved to “no” at post-read (Table 5).

**Table 5.** Do you think that you would ever want to be part of a research study? Pre/Post comparison of online vs. In-person

		Post-Read Response			
Modality	Pre-Read Response	No	Not Sure	Yes	Total
Online (*n* = 47)	No	3 (60%)	**1 (20%)**	**1 (20%)**	5
	Not Sure	2 (11.1%)	13 (72.2%)	**3 (16.7%)**	18
	Yes	0 (0.0%)	0 (0.0%)	**24 (100.0%)**	24
In-Person (*n* = 34)	No	3 (42.9%)	**4 (57.1%)**	0 (0%)	7
	Not Sure	3 (16.7%)	10 (5.6%)	**5 (27.8%)**	18
	Yes	2 (22.2%)	3 (33.3%)	4 (44.4%)	9

## Conclusions

This child and family-friendly informational tool aims to increase awareness and knowledge about research. We have shown that it is feasible to disseminate the book in both passive and active online and in-person modalities (PB-E vs. DI-E). We piloted strategies to evaluate knowledge and interest in participating in research pre and post book exposure. Our findings suggest that “Sofia Learns About Research” could be effective at increasing the reader’s knowledge of assent and consent and the ability that the participant can opt out of research even after they are enrolled. The preliminary findings also point to the book’s potential to help the reader understand that health research “is performed in order to improve people’s health.”

We observed differences in how knowledge and intent changed by age and modality of book implementation. These comparisons are limited by small sample size and variation in the in-person and online implementation methods. The in-person sessions were interactive and prompted dialog between teachers/researchers and readers. They were delivered by teachers or different members of the research team, ranged in duration, and varied in the discussion prompts. Online reading experiences were either initiated by a teacher as part of their science curriculum during COVID lockdown or were invitations through a research registry. In both online methods, most of the onus of understanding was on the reader, leaving them responsible for completing the readings and surveys, and relying on their ability of them to learn independently. For the purposes of this report, we dichotomously grouped all online and all in-person, but plan to disaggregate by these additional characteristics when we have more data.

Results suggest that the online modality was more effective at keeping people at “yes” as well as moving people to “yes” in response to the question “Do you think that you would ever want to be part of a research study?” However, age may also play a role, as the online participants were older, on average than the in-person participants. Online participants also included people who were signed up with a research registry and thus are already open to participation in research.

When determining the book’s impact on youth knowledge and interest in participating in research, there were inconsistencies in participant responses by age. There were some statements that were answered incorrectly at post-read after being answered correctly at pre-read, particularly by the younger respondents. This could mean that either the statements were confusing to children, or the book did not explain those concepts effectively, especially in ways younger children could understand. Indeed, it is possible the book and its one-time reading may not be enough to increase knowledge and interest in participation in research.

Since the start of book development in 2016, several other similar tools have emerged at other U.S. institutions [13–15]. This is evidence of the need to better explain research to an underrepresented population in research as well as the parallel evolution and implementation of child and family-friendly approaches. It is difficult to convey the potential benefits of involvement in research in a child-friendly way while maintaining the awareness of the potential risks as well as choice in all aspects of participation. For example, by positioning someone who participates in research as a Research Ranger superhero who helps solve problems and improves children’s health, there is a potential risk of coercing or applying undue influence on a child to participate in research. Our strategy to address this included the conversation between Sofia and the researcher, where Sofia asked about whether she could stop participating in the study. The researcher answers this in three different phrases (“you can stop at anytime,” “if you decide that you do not want to do it anymore, that’s okay,” and “you are allowed to stop whenever you want”). This is an attempt to demonstrate trust and transparency in communication, an area we would like to explore further.

In addition to revising the knowledge questions to address some of the limitations with younger audiences and adding measures of trust, we also plan to further evaluate how different implementation modalities may impact knowledge and interest differently. Though our current data does not demonstrate this, it is our observation that in-person interactive sessions, ranging from 2–4 hours in total, have the greatest potential to increase youth knowledge and awareness of research. Changing interest or intention to participate, though, may require more time and additional strategies. Online dissemination may be an acceptable approach to populations already familiar with research, such as members of a research registry, or youth who have additional support from an actively engaged teacher or other facilitator. The online version is accessible on computers, tablets, and smartphones. Recent local estimates show that most households have access to online environments through smartphones.

Future directions include continued outreach and revised evaluation within schools, pediatric health care centers, afterschool programs, and libraries. To ensure we are framing this as research “with” children, not “on” children [16–18], this will include conversations with youth and people who work with youth about the readability and clarity of the questions in the pre/post survey. A potential modification includes moving the interactive activities to the end of the book so as not to disrupt the flow of the story. A revised version of the book will be used in the second phase of this project where we aim to test the impact of the book on actual participation in research. Researchers have expressed interest in testing the use of the book in conversations where children and their parents are invited to participate in a study and proceed through the informed consent process. Edits to the book will continue to carefully consider research ethics and be responsive to child developmental stages.

It is with this spirit that as of August 2024, over 3,000 books have been printed and disseminated to multiple locations and organizations, including pediatric clinics, schools, libraries, Roswell Park Comprehensive Cancer Center, Rochester CTSA Hub, and the International Pediatric Stroke Organization’s online community publication. Additionally, our partners at Walgreens Clinical Trials distributed 100 Sofia books at the “Friends for Life Orlando 2023 - Children with Diabetes” (over 1,800 families in attendance) and an additional 100 during National Rural Health Day in Monroe, GA.
